# Mannose-Functionalized Polymeric Micelles Target CD206-Expressing Macrophages Within Human Tuberculosis Granulomas and Improve Isoniazid Delivery

**DOI:** 10.3390/pharmaceutics18101279

**Published:** 2026-10-09

**Authors:** Camila Belen Martinena, Facundo Guillermo Ortellado, Agustín Daniel Vitti, Candela Martin, Ana Julia Bazán Bouyrie, Ezequiel Bernabeu, Rocío Zuazo, Diego Andrés Chiappetta, Verónica Edith García, Nicolas Oscar Amiano, Marcela Analia Moretton, Nancy Liliana Tateosian

**Affiliations:** 1 Instituto de Química Biológica, Facultad de Ciencias Exactas y Naturales (IQUIBICEN), Universidad de Buenos Aires (UBA)-Concejo Nacional de Investigaciones Cientificas y Tecnicas (CONICET), Intendente Güiraldes 2160, Pabellón II, 4°piso, Ciudad Universitaria, Buenos Aires C1428EGA, Argentina; cami.b.martinena@gmail.com (C.B.M.); vittiagustin@gmail.com (A.D.V.); cande.martiin@gmail.com (C.M.); rociozuazo@gmail.com (R.Z.); vgarcia@qb.fcen.uba.ar (V.E.G.); nico.amiano@qo.fcen.uba.ar (N.O.A.); 2 Departamento de Química Biológica, Facultad de Ciencias Exactas y Naturales, Universidad de Buenos Aires, Intendente Güiraldes 2160, Pabellón II, 4°piso, Ciudad Universitaria, Buenos Aires C1428EGA, Argentina; fortellado7@gmail.com (F.G.O.); ana.bazan19@gmail.com (A.J.B.B.); 3 Facultad de Farmacia y Bioquímica, Universidad de Buenos Aires, Junin 956, Buenos Aires C1113AAD, Argentina; eze_bernabeu@yahoo.com.ar (E.B.); diegochiappetta@yahoo.com.ar (D.A.C.); marcelamoretton@gmail.com (M.A.M.); 4 Instituto de Tecnología Farmacéutica y Biofarmacia (InTecFyB), Universidad de Buenos Aires, Junin 956, Buenos Aires C1428EGA, Argentina; 5 Consejo Nacional de Investigaciones Cientificas y Tecnicas (CONICET), Godoy Cruz 2290, Buenos Aires C1425FQB, Argentina; 6 Departamento de Química Orgánica, Facultad de Ciencias Exactas y Naturales, Universidad de Buenos Aires, Intendente Güiraldes 2160, Pabellón II, 3°piso, Ciudad Universitaria, Buenos Aires C1428EGA, Argentina

**Keywords:** latent tuberculosis, *Mycobacterium tuberculosis*, mannose targeting, CD206 expressing macrophages, polymeric micelles, granuloma model, intracellular drug delivery

## Abstract

**Background**: Latent tuberculosis infection (LTBI) remains a global health challenge, in part due to the ability of *Mycobacterium tuberculosis* (*Mtb*) to persist within granulomatous lesions where drug exposure may be limited. Because granulomas are heterogeneous structures composed of different macrophage populations, targeting disease-relevant intracellular niches may improve antimicrobial treatment. Here, we developed mannose-functionalized polymeric micelles loaded with isoniazid (INH) to target mannose receptor (CD206)-expressing macrophages within a human tuberculosis granuloma model. **Methods**: Mannose-functionalized Soluplus^®^ micelles loaded with INH (ManPM-INH) and non-functionalized micelles (PM-INH) were characterized for particle size, polydispersity, and colloidal stability. Antimycobacterial activity against *Mtb*H37Rv was evaluated by CFU assays. A human peripheral blood mononuclear cell (PBMC) three-dimensional in vitro granuloma model was used to assess micelle accumulation and antimycobacterial activity. Cellular uptake was evaluated in M0, M1, and M2 macrophages using rhodamine-labeled micelles. CD206-associated and clathrin-mediated uptake were investigated using D-mannose and chlorpromazine, respectively. **Results**: ManPM-INH exhibited suitable physicochemical properties and maintained colloidal stability after mannose functionalization. In the granuloma model, ManPM showed significantly greater intracellular accumulation than PM. M2-like macrophages predominated within granulomas, and ManPM displayed preferential uptake by M2 macrophages. Furthermore, D-mannose competitively reduced ManPM uptake in macrophages with significantly greater inhibition in M2 cells. Chlorpromazine reduced ManPM internalization, supporting involvement of a clathrin-dependent pathway. ManPM-INH achieved the greatest reduction in intracellular *Mtb*H37Rv burden compared with INH and PM-INH, particularly at low drug concentrations. **Conclusions**: Mannose-functionalized micelles enhance intracellular INH delivery by targeting CD206-expressing macrophages within tuberculosis granuloma, supporting macrophage-targeted nanotherapeutic strategies against persistent intracellular *Mtb*.

## 1. Introduction

Tuberculosis (TB), caused by *Mycobacterium tuberculosis* (*Mtb*), remains one of the leading infectious causes of death worldwide. According to the World Health Organization, approximately 10.7 million people developed TB and more than one million died from the disease in 2024. Despite the availability of effective chemotherapy, TB control continues to be hindered by the large reservoir of latent tuberculosis infection (LTBI), which is estimated to affect nearly one-quarter of the global population [1]. Although individuals with LTBI are asymptomatic and non-contagious, viable bacilli may persist within host tissues and reactivate years after primary infection. It is estimated that 5–10% of immunoreactive individuals will eventually develop active TB during their lifetime, making LTBI a major contributor to the sustained global burden of infection [2].

Following inhalation of infectious aerosols, *Mtb* is phagocytosed by alveolar macrophages, while dendritic cells contribute to the initiation of adaptive immune responses [3]. The subsequent recruitment of macrophages, monocytes, lymphocytes, and other immune cells to the site of infection leads to the formation of granulomas, the pathological hallmark of TB [4]. These highly organized structures restrict bacterial dissemination while simultaneously providing a protected microenvironment in which *Mtb* persists in a metabolically quiescent, non-replicating, drug-tolerant state [4,5].

Among the diverse cellular populations present within granulomas, macrophages represent the primary intracellular host cells for *Mtb* and play a central role in bacterial persistence [6]. These cells exhibit remarkable phenotypic plasticity in response to local inflammatory and metabolic signals [7]. Although macrophage activation is increasingly recognized as a dynamic spectrum rather than discrete activation states, the classical M1/M2 paradigm remains a useful framework for describing their predominant functional phenotypes [8,9]. M1-like macrophages are generally associated with enhanced antimicrobial effector functions, whereas M2-like macrophages contribute to tissue remodeling, immune regulation, and resolution of inflammation [8,10]. Macrophage heterogeneity within TB granulomas has become increasingly recognized as a critical determinant of disease progression and therapeutic outcome. Multiple macrophage phenotypes coexist within granulomatous lesions, including inflammatory, epithelioid, multinucleated giant, foamy, and alternatively activated macrophages, creating distinct intracellular niches that differ in their metabolic activity, antimicrobial capacity, and susceptibility to drug exposure [11]. This cellular diversity represents both a challenge and an opportunity for the development of macrophage-targeted drug delivery strategies. Among these populations, M2 macrophages with high expression of the mannose receptor CD206 have emerged as potential targets for macrophage-directed drug delivery, given their role in bacterial persistence and the ability of CD206 to mediate the uptake of mannose-containing ligands [12,13].

Although granulomas are essential for containing infection, they also constitute the principal pharmacological barrier to effective chemotherapy by limiting antimicrobial penetration and drug exposure within the lesion [5,14]. Current preventive treatment of LTBI relies primarily on isoniazid (INH) monotherapy or rifamycin-based regimens [15]. Although these therapies reduce the risk of progression to active TB, their effectiveness is often limited by prolonged treatment duration, drug-related toxicity, and incomplete treatment adherence [16]. Moreover, because *Mtb* bacilli can persist within macrophage-rich granulomatous lesions, where drug penetration is heterogeneous and the local microenvironment can favor bacterial persistence, achieving adequate drug exposure at the site of infection remains a major therapeutic challenge [5,17]. These limitations underscore the need for advanced drug delivery systems capable of enhancing drug accumulation within granulomatous lesions, prolonging intracellular retention, and improving antimicrobial exposure at sites of bacillary persistence. Among these approaches, nanotechnology-based drug delivery systems have emerged as promising strategies to overcome the pharmacological barriers imposed by granulomatous tuberculosis. By enhancing drug accumulation at the site of infection, nanocarriers can increase local drug exposure, prolong intracellular drug retention, promote uptake by infected macrophages, and reduce systemic toxicity [18]. Among the different nanocarrier platforms, polymeric micelles have attracted considerable interest owing to their biocompatibility, colloidal stability, and ease of surface functionalization. Previously, we demonstrated that mannose functionalization significantly improved the microbicidal efficacy of rifampicin-curcumin-loaded polymeric micelles against *Mtb* H37Rv-infected macrophages [19]. Building on these findings, the present study focuses on the interaction of mannose-functionalized polymeric micelles with distinct macrophage phenotypes within the heterogeneous environment of a human tuberculosis granuloma. In particular, we investigated whether the differential expression of CD206 among macrophage phenotypes is associated with differences in the uptake of mannose-functionalized micelles and evaluated the contribution of mannose-dependent and clathrin-associated uptake mechanisms. In the present study, we hypothesize that preferential expression of CD206 by alternatively activated macrophage populations located within the granuloma core [20] would enhance intracellular delivery of mannose-functionalized polymeric micelles, increasing INH exposure of persistent *Mtb*. Indeed, the effectiveness of targeted nanocarriers depends not only on ligand–receptor interactions but also on the presence and distribution of the target cell population within the infected tissue microenvironment. Accordingly, we employed Soluplus^®^, a graft copolymer with excellent colloidal properties that has been successfully used in micellar nanoformulations for antiviral and antifungal therapy [21,22,23], to evaluate whether macrophage-targeted nanocarriers can achieve selective intracellular delivery under infection-like conditions. To this end, we employed a human PBMC-derived three-dimensional (3D) granuloma model that recapitulates key features of latent tuberculosis, preserving key features of tuberculosis-associated cellular heterogeneity. We evaluated the uptake of isoniazid-loaded mannose-functionalized polymeric micelles by different macrophage phenotypes within the granuloma and investigated whether mannose functionalization was associated with increased intracellular accumulation and antimycobacterial activity against persistent intracellular *Mtb*.

## 2. Materials and Methods

### 2.1. Micellar Preparation and Drug Encapsulation

Soluplus^®^ (1 g) was dispersed in distilled water (10 mL) under magnetic stirring (50 rpm for 1 h) at room temperature (25 °C) to obtain empty polymeric micelles (PM). The dispersion was stored overnight at room temperature before further use. Mannose-functionalized PM were obtained by the addition of mannose-modified gelatin (Gel(man)) (50 mg) under continuous magnetic stirring (50 rpm) until complete and homogeneous dispersion was achieved, as previously reported [19]. Gel(man) was obtained from dried bovine gelatin and mannose with an estimated mannose ratio in the modified Gel of 4.2% [19]. Furthermore, we employed a blend of Soluplus^®^/Gel(man) (10 and 0.5% *w*/*v*) as previously studied [19].

For the preparation of INH-loaded formulations, the drug (INH, 10 mg, Sigma-Aldrich, St. Louis, MO, USA) was incorporated into the aqueous micellar dispersions containing Soluplus^®^ alone or Soluplus^®^/Gel(man) under constant magnetic stirring (50 rpm) at room temperature for 30 min. After drug incorporation, the final volume of the formulations was adjusted to 10 mL with distilled water, and the dispersions were filtered (0.45 µm, cellulose acetate filters, Microclar, Buenos Aires, Argentina). The resulting formulations were designated as INH-loaded polymeric micelles (PM-INH) and mannose-functionalized INH-loaded polymeric micelles (ManPM-INH). INH quantification for every micellar formulation was performed by UV-Visible spectroscopy (263 nm, CARY [1E] UV–Visible Spectrophotometer, Varian, Palo Alto, CA, USA) at 25 °C using a calibration curve of INH solutions in methanol covering the range between 2 and 10 μg/mL (correlation factor was 0.9997–0.9999).

Rhodamine B isothiocyanate (RhB)-loaded micelles were prepared using the solvent diffusion method, as previously described [19]. Briefly, RhB (10 mg, Merck, Darmstadt, Germany) was dissolved in acetone (5 mL), sonicated for 5 min (Digital Ultrasonic Cleaner, PS-10A, 50/60 Hz, Shenzhen, China) at 25 °C, and added dropwise to an aqueous Soluplus^®^ dispersion (10% *w*/*v*) using a programmable syringe infusion pump (PC11UB, APEMA, Buenos Aires, Argentina) under continuous magnetic stirring (50 rpm) at room temperature. The system was maintained under stirring for 4 h to allow complete evaporation of the organic solvent. The final volume of RhB-loaded polymeric micelles (PM-RhB) was adjusted to 10 mL with distilled water, and the samples were filtered through 0.45 µm cellulose acetate filters to remove undissolved and/or large RhB aggregates. Mannose-functionalized RhB-loaded polymeric micelles (ManPM-RhB) were prepared following the same procedure with minor modifications. After complete evaporation of acetone, Gel(man) (50 mg) was added under magnetic stirring at 25 °C until complete homogenization. The resulting formulation was subsequently processed as described above.

Finally, all micellar formulations were freeze-dried for 24 h at 0.03 mbar using a freeze dryer (FIC-L05, FIC Scientific Instrumental Manufacturing, Buenos Aires, Argentina) and stored in sealed amber glass vials at room temperature until further use.

### 2.2. Characterization of the Micellar Dispersions

The hydrodynamic diameter and polydispersity index (PDI) and zeta potential of both PM-INH and ManPM-INH were determined by Dynamic Light Scattering (DLS) using a Zetasizer Nano-ZS instrument (Malvern Instruments, Malvern, UK), equipped with a 4 mW He–Ne laser operating at 633 nm and a ZEN3600 digital correlator. Measurements were performed at 25 °C with a detection angle of 173° relative to the incident laser beam. Prior to analysis, micellar dispersions were prepared in Milli-Q water, passed through 0.45 μm cellulose nitrate clarifying filters, and allowed to equilibrate at 25 °C for a minimum of 30 min. The experimental data were processed using the CONTIN algorithm implemented in the Malvern Instruments software version 7.11. The Z-average (Z-ave) and PDI values are reported as the mean of at least three independent measurements. Furthermore, PM-INH and ManPM-INH nanoformulations were visualized by means of transmission electron microscopy (Philips CM-12 TEM apparatus, FEI Company, Eindhoven, The Netherlands). Briefly, 5 μL of each micellar dispersion was deposited onto a Formvar-coated grid and allowed to stand for 30 s. Excess liquid was then removed using filter paper. The grids were subsequently stained with 2% (*w*/*v*) uranyl acetate for 30 s, followed by removal of the excess staining solution. A 5 μL aliquot of water was then added to the grid, maintained for 30 s, and carefully removed. The prepared samples were dried in a closed container containing silica gel and subsequently examined by TEM.

### 2.3. In Vitro Micellar Stability

The physicochemical stability of the nanoformulations was investigated under in vitro conditions before and after nebulization [24]. The formulations were evaluated following dilution in either 0.9% (*w*/*v*) NaCl solution or distilled water. Briefly, freeze-dried PM-INH and ManPM-INH containing 1 mg/mL of INH were reconstituted in 2 mL of distilled water to get a final Soluplus^®^/Gel(man) concentration of 10 and 0.5% *w*/*v*, respectively. The resulting dispersions were subsequently diluted fivefold with either normal saline solution or distilled water. Nebulization was then performed for 5 min using a Maverick Ultrasonic Nebulizer Mesh (NE-M01L, Contec Medical Systems Co., Ltd., Qinhuangdao, China). The aerosol generated was recovered in a volumetric glass flask coupled to the nebulizer.

To assess the effect of nebulization on the formulations, Z-ave, size distribution, and PDI were determined by DLS immediately before and after nebulization at 25 °C, according to the procedure described above. All measurements were performed in triplicate, and the results are reported as Mean ± standard deviation (S.D.).

### 2.4. Cell Isolation from Human Blood and Macrophage Differentiation

Human blood samples were obtained from healthy donors after written informed consent. Peripheral blood mononuclear cells (PBMCs) were isolated by Ficoll-Hypaque density gradient centrifugation (Cytiva, Marlborough, MA, USA). PBMCs were subsequently used for either in vitro granuloma formation or monocyte isolation by hyper-osmotic Percoll density gradient centrifugation as described [25]. Monocyte purity (>85%) and viability (>95%) were confirmed by flow cytometry and trypan blue exclusion, respectively.

Purified monocytes were differentiated into macrophages by culture with GM-CSF (50 ng/mL, Cassará, Buenos Aires, Argentina) for 5–7 days. Macrophages were subsequently polarized toward an M1-like phenotype by stimulation with IFN-γ (10 ng/mL, BioLegend, San Diego, CA, USA) and *Mtb* antigen (10 µg/mL, Bei Resources, Manassas, VA, USA), whereas M2-like macrophages were generated by stimulation with IL-4 (40 ng/mL, BioLegend, San Diego, CA, USA) and IL-13 (20 ng/mL, BioLegend, San Diego, CA, USA). Macrophage polarization was confirmed by flow cytometry.

For uptake experiments, macrophages were seeded in glass-bottom 24-well plates at a density of 8 × 10^4^ cells/well and incubated with PM-RhB or ManPM-RhB. When indicated, cells were pretreated with chlorpromazine (CPZ; 15 µM, Cayman chemicals, Ann Arbor, MI, USA) to inhibit clathrin-mediated endocytosis or with D-mannose (50mM, Santa Cruz Biotechnology Inc., Dallas, TX, USA) for CD206 competition before exposure to micellar formulations. Cell viability under these experimental conditions was assessed using the CyQUANT™ XTT Cell Viability Assay (Invitrogen, Carlsbad, CA, USA).

### 2.5. Mycobacterial Culture Conditions

*Mycobacterium tuberculosis* H37Rv (*Mtb*H37Rv) and the attenuated strain *M. tuberculosis* mc26230 (ΔRD1 ΔpanCD; *Mtb*ΔRD1) were cultured in Middlebrook 7H9 broth or on Middlebrook 7H10 agar supplemented with 0.2% glycerol and oleic acid–albumin–dextrose–catalase (OADC; BD Biosciences, San José, CA, USA, 10% *v*/*v*). Bacterial cultures were harvested during the exponential growth phase at 37 °C. To disrupt bacterial aggregates, mycobacteria were sonicated at 2.5 W for 4 min (Elma D-7700, Singen, Germany) and centrifuged at 2000 rpm for 10 min. The supernatant was collected, diluted in 7H9 broth containing glycerol and OADC, and bacterial density was determined by measuring optical density at 600 nm. All experiments involving virulent *Mtb*H37Rv were performed under biosafety level 3 (BSL-3) conditions at the ANLIS-Malbrán Institute (Buenos Aires, Argentina).

### 2.6. Antimycobacterial Activity in Axenic Cultures

*Mtb*H37Rv cultures (1 × 10^6^ CFU/100 µL) were incubated in Middlebrook 7H9 broth supplemented with glycerol and OADC and treated with PM-INH or ManPM-INH at final INH concentrations of 0.005, 0.01, and 0.05 µg/mL for 48 h or 7 days. Antimycobacterial activity was evaluated by colony-forming unit (CFU) enumeration.

### 2.7. Human In Vitro Granuloma Formation

The human in vitro TB granuloma model was generated based on a previously described protocol with modifications [26,27]. PBMCs were infected with *Mtb*H37Rv or *Mtb*ΔRD1 at a multiplicity of infection (MOI) of 0.005 and seeded in 24-well plates at a concentration of 1 × 10^7^ cells/mL. An extracellular matrix (ECM) solution was prepared by mixing gelatin from bovine skin (Sigma-Aldrich, St. Louis, MO, USA), fibronectin 1mg/mL (Invitrogen, Carlsbad, CA, USA) and 1 N NaOH (Carlo Erba Reagents, Milan, Italy) in PBS 10X. The ECM solution was mixed with infected PBMCs at a 1:1 ratio (*v*/*v*) and incubated at 37 °C for 45 min under 5% CO_2_ to allow matrix formation. After ECM polymerization, wells were supplemented with 500 µL of RPMI-1640 medium (Gibco, Grand Island, NY, USA) containing 10% fetal bovine serum (Natocor, Córdoba, Argentina) and L-glutamine (2 mM; Gibco, Grand Island, NY, USA), and cultures were maintained at 37 °C under 5% CO_2_.

Granuloma formation was monitored on days 7–8 post-infection by inverted microscopy using an Olympus microscope (Olympus Corporation, Tokio, Japan). At day 7, granulomas were treated with: (i) freeze-dried PM-INH or ManPM-INH formulations for 48 h or 7 days at final INH concentrations of 0.005, 0.01, and 0.05 µg/mL; (ii) freeze-dried PM-RhB or ManPM-RhB formulations for 2, 4, 6, 24, or 48 h at a final RhB concentration of 0.5 µg/mL.

For Colony-Forming Unit Assay (CFU) determination and Auramine O/Nile Red staining, granulomas were mechanically disrupted and cells were lysed with 0.05% Triton X-100 in PBS. For confocal microscopy, granulomas were fixed with 4% paraformaldehyde prior to imaging.

### 2.8. Colony-Forming Unit Assay

Serial dilutions of *Mtb*H37Rv cultures or granuloma cell lysates were prepared, and 10 µL aliquots were plated in triplicate on Middlebrook 7H10 agar supplemented with OADC (10% *v*/*v*). Plates were incubated for approximately 3 weeks, and colonies were counted from dilutions containing 10–100 colonies.

### 2.9. Confocal Microscopy

All cells were fixed with 4% paraformaldehyde prior to imaging. Cell nuclei were stained with DAPI (Cell Signaling, Danvers, MA, USA). The coverslips were mounted with Mowiol (Sigma-Aldrich, St. Louis, MO, USA) overnight. Cells displaying preserved morphology were selected based on bright-field imaging. Images were acquired using confocal laser scanning microscopy (LSM980, ZEISS, Jena, Germany) equipped with a 63× oil immersion objective. DAPI was excited at 405 nm, whereas RhB fluorescence was excited at 543 nm. For granuloma imaging, z-stack images were obtained with optical sections of 3 µm thickness.

Confocal microscopy images were analyzed using ImageJ (Fiji) software version 1.54. RhB-positive cells were identified by applying an intensity threshold to the rhodamine channel, generating a binary mask to define individual cell regions of interest (ROIs). Rhodamine fluorescence was quantified as mean fluorescence intensity (MFI) for each ROI. Background fluorescence was measured in cell-free regions of the same optical section and subtracted from the corresponding cellular fluorescence measurements.

For M0, M1, and M2 macrophages, a minimum of 10 cells per macrophage phenotype and experimental condition were analyzed for each healthy donor. MFI values from individual cells were averaged within each donor, with each donor considered one biological replicate (N = 6). For granuloma analysis, two representative optical sections (z-slices) were selected from each granuloma. Thus, the MFI per granuloma represents the average cellular fluorescence within the analyzed optical sections rather than the cumulative fluorescence of the entire granuloma. Three granulomas per healthy donor were analyzed for each experimental condition and time point.

### 2.10. In Vitro Cytotoxicity Study

For the in vitro cytotoxicity assays, PBMCs were seeded in 96-well plates at a density of 100,000 cells/well. Then, cells were incubated with five different concentrations (0.05, 0.025, 0.012, 0.006, 0.003 µg/mL) of PM-INH and ManPM-INH for 48 h and 7 days. Also, different concentrations of empty micelles were assayed as a control. After incubation, the medium was removed, and fresh medium was added. Then, we followed the CyQUANT™ XTT Cell Viability Assay kit manufacturer’s instructions (Cat No X12223, Invitrogen, Carlsbad, CA, USA). Briefly, the XTT working solution was added to each well, and cells were incubated for 4 h at 37 °C. The absorbance at 450 nm and 660 nm was measured using a microplate reader (DR-200Bs Microplate Reader, Wuxi Hiwell Diatek Instruments Co., Wuxi, China). Specific absorbance was calculated as indicated in the manufacturer’s instructions. Triplicates were run for each treatment. Values were expressed in terms of percent of untreated control cells set as 100%. Cell viability was interpreted according to the criteria established in ISO 10993-5:2009, which defines materials as non-cytotoxic when cell viability remains above 70% relative to untreated controls [28].

### 2.11. Flow Cytometry

PBMCs from disaggregated in vitro granulomas were stained with specific fluorophore-marked antibodies against CD14 (FITC, Biolegend, San Diego, CA, USA). Cells were stained with HLA-DR antibody (PeCy7, Biolegend, San Diego, CA, USA) and CD80 (PE, Biolegend, San Diego, CA, USA) to detect M1-like polarization or with anti-CD206 (PE, Biolegend, San Diego, CA, USA) and anti-DC-SIGN (APC, BD Biosciences, San José, CA, USA) for M2-like detection. Upon purification, macrophages were stained with M1 or M2 markers. Negative control samples were incubated with irrelevant isotype-matched monoclonal antibodies (Biolegend, San Diego, CA, USA). Samples were analyzed on a FACSAria II flow cytometer (BD Biosciences, San José, CA, USA).

### 2.12. AuramineO-Nile Red Staining

To determine the latent phenotype of *Mtb*, about 20 µL of culture at different time points were evenly spread to make a thin smear on a glass slide, quickly heat-fixed oVER a flame and cooled to room temperature before staining. Each smear was covered with Auramine-O (10 µg/mL, Biopack, Buenos Aires, Argentina), incubated for 20 min, gently washed with distilled water, treated with decolorizer solution for 30 s, washed with distilled water, covered with the second dye, Nile Red solution (10 µg/mL in acetone), incubated for 15 min, washed with water, covered with potassium permanganate solution for 1 min, washed thoroughly with water and air-dried. The staining procedure was carried out in a BSL3 hood without direct light exposure. Stained smears were mounted using Mowiol and thin cover glass (less than 150 micron thick). The mounted slides were dried in the dark for at least 24 h before examination under a confocal laser scanning microscope (LSM980, ZEISS, Jena, Germany). The confocal scanning images were analyzed and projected using ImageJ.

### 2.13. Statistical Analysis

Statistical analyses of CFU assays were performed using one-way analysis of variance (ANOVA) followed by Tukey’s multiple comparison test. Time kinetics of uptake inside in vitro granuloma were analyzed using Two-way ANOVA with post hoc Tukey’s multiple comparisons test. For micelle uptake experiments, statistical analysis was performed using a one-way repeated-measures ANOVA with Greenhouse–Geisser correction, followed by Holm–Šidák multiple-comparisons test. M1 and M2 markers in M0/M1/M2 phenotype data were analyzed using repeated-measures ANOVA (with Greenhouse–Geisser correction for baseline donor variability). Comparisons of the percentage inhibition of micelle uptake between M1 and M2 macrophages were performed using a paired Student’s *t*-test. Differences were considered statistically significant when *p <* 0.05.

## 3. Results

### 3.1. Physicochemical Characterization of Mannose-Functionalized Soluplus^®^ Polymeric Micelles for INH Delivery

INH-loaded polymeric micelles (PM-INH) and mannose-functionalized INH-loaded polymeric micelles (ManPM-INH) nanoformulations were successfully prepared, exhibiting physicochemical characteristics suitable for pulmonary drug delivery. As shown in Table 1, DLS analysis confirmed that both formulations were within the nanoscale range and displayed relatively narrow size distributions. PM-INH exhibited Z-ave values of 115.1 ± 1.9 nm, with a PDI of 0.214 ± 0.003 (Table 1). In the case of ManPM-INH, the Z-ave increased to 166.5 ± 1.5 nm, with a PDI of 0.403 ± 0.013 (Table 1). Functionalization of the micellar surface with Gel(man) resulted in an increase in hydrodynamic diameter, suggesting that mannose functionalization did not compromise the colloidal homogeneity of the system. This increase in hydrodynamic diameter may be associated with the hydrophilic nature of the polyethylene glycol (PEG) corona of the micelles and its interactions with the hydrophilic Gel(man) layer, which may affect the overall hydrodynamic size of the micelles. A similar behavior has previously been reported by our research group for Soluplus^®^/Gel(man)-based micellar formulations loaded with rifampicin and curcumin [19]. Additionally, we have previously demonstrated the surface location of the mannose moieties employing a water-soluble lectin (Concanavalin A) assay [19], and zeta potential values for both nanoformulations, in the presence (0.648 ± 0.682 mV) and absence of Gel(man) (0.025 ± 0.061 mV), showed that the micellar surface was almost neutral.

To improve their medium- and long-term stability, the nanoformulations were subsequently subjected to freeze-drying. Although lyophilization is widely used to enhance the storage stability of nanosized systems, the process may promote particle aggregation and compromise colloidal stability upon reconstitution [29]. In the present study, both INH nanoformulations could be successfully lyophilized without the addition of cryo- or lyoprotective agents, and no visible signs of powder collapse were observed (Figure 1A). Following reconstitution in distilled water, the dispersions remained visually clear, with no macroscopic aggregates detectable by the naked eye. DLS measurements further confirmed the preservation of colloidal stability after reconstitution. PM-INH exhibited Z-ave values of 118.3 ± 3.4 nm, which were comparable to those determined before lyophilization (Table 1). Similar behavior has been previously reported for other Soluplus^®^-based micellar formulations [23,30]. Interestingly, lyophilization resulted in a reduction in the hydrodynamic diameter of ManPM-INH, from 166.5 ± 1.5 nm before freeze-drying to 131.2 ± 0.2 nm after reconstitution, while maintaining a relatively narrow size distribution (Table 1). This decrease in micellar size may be related to a contraction or rearrangement of the micellar structure during the lyophilization process, potentially arising from the dynamic nature of polymeric micelles [31,32].

The morphology of the nanoformulations was further assessed by transmission electron microscopy (TEM). As shown in Figure 1B,C, the representative TEM images of PM-INH and ManPM-INH showed a predominantly spherical morphology, consistent with the formation of nanosized micellar structures. The apparent differences in particle size between TEM and DLS measurements may be attributed, at least in part, to dehydration during TEM sample preparation, which can alter polymer self-aggregation and, consequently, the observed particle size [33,34].

Considering the dynamic nature of PM, the colloidal stability of the INH pulmonary nanoformulations was further investigated following nebulization. For this purpose, the freeze-dried formulations were first redispersed in distilled water and subsequently diluted five-fold with either (i) 0.9% (*w*/*v*) NaCl solution or (ii) distilled water, conditions intended to simulate the formulation environment during pulmonary administration using a nebulizer device. As shown in Table 2, both micellar formulations exhibited excellent colloidal stability after nebulization. When diluted in normal saline solution, all INH systems showed a narrow size distribution, with PDI values below 0.09, irrespective of the presence of the Gel(man) coating. A similar behavior was observed when the formulations were diluted in distilled water, with no relevant changes in hydrodynamic diameter or size distribution in the size distribution following nebulization (Table 2). These results indicate that neither the dilution medium nor the nebulization process substantially affected the colloidal characteristics of PM-INH and ManPM-INH.

### 3.2. Antimycobacterial Activity of INH-Loaded Soluplus^®^ Micelles Against Axenic Mtb Culture

To investigate whether mannose functionalization affected the intrinsic antimycobacterial activity of INH, axenic cultures of *Mtb*H37Rv were exposed to soluble INH, PM-INH or ManPM-INH at concentrations of 0.005, 0.01, and 0.05 µg/mL for 48 h or 7 days (Figure 2). This concentration range was selected based on preliminary baseline screening assays, in which the MIC values were determined to be 0.05 µg/mL for free INH, 0.025 µg/mL for PM-INH, and 0.012 µg/mL for ManPM-INH, thereby providing a concentration profile spanning sub-MIC to MIC/near-MIC conditions across the different formulations. Because these experiments were performed in the absence of host cells, they specifically assessed whether micellar formulation and surface mannosylation altered the direct bactericidal activity of INH. After 48 h of treatment (Figure 2A), both micellar formulations exhibited enhanced antimycobacterial activity compared with soluble INH, particularly at suboptimal drug concentrations. At 0.005 and 0.01 µg/mL, PM-INH and ManPM-INH were more effective than free INH, whereas no significant differences were detected between the two micellar formulations. At 0.05 µg/mL, all treatments markedly decreased bacterial viability; however, both micellar formulations remained significantly more active than soluble INH, indicating that encapsulation preserves the antimicrobial performance of the drug under axenic conditions. Following 7 days of treatment (Figure 2B), the superiority of the micellar formulations over soluble INH became evident at the two lowest concentrations tested. In contrast, treatment with 0.05 µg/mL resulted in an almost complete inhibition of bacterial growth in all groups, with no significant differences among formulations, consistent with the achievement of maximal bactericidal activity at this concentration. Overall, these findings demonstrate that surface functionalization with mannose does not compromise the intrinsic antimycobacterial activity of encapsulated INH. Instead, the bactericidal activity of the drug was fully preserved after functionalization and remained comparable or superior to that of non-functionalized micelles across the concentrations evaluated.

### 3.3. Biocompatibility of Polymeric Micellar Formulations in Human PBMCs

Before investigating nanoparticle uptake and antimycobacterial efficacy in the human 3D granuloma model, the cytocompatibility of the different micellar formulations was evaluated in PBMCs using the XTT assay (Figure S1). PBMCs were incubated with empty polymeric micelles (PM), empty mannosylated polymeric micelles (ManPM), PM-INH, or ManPM-INH at concentrations ranging from 0.003 to 0.05 μg/mL for either 48 h or 7 days. All formulations exhibited excellent cytocompatibility under the experimental conditions evaluated. Cell viability remained above the 70% threshold for all treatments, regardless of drug loading, mannose functionalization, or incubation time. According to the criteria established by ISO 10993-5, materials maintaining cell viability above 70% are considered non-cytotoxic, indicating that neither INH encapsulation nor mannose surface functionalization adversely affected PBMC viability. These findings demonstrate that all micellar formulations were well tolerated by human immune cells at the concentrations employed throughout the study and therefore were suitable for subsequent evaluation in the human in vitro 3D granuloma model.

### 3.4. Mannose-Functionalized Micelles Enhance Intracellular Accumulation Within the Human TB Granuloma Model

To determine whether mannose functionalization enhances micelle uptake within the granuloma, uptake kinetics of PM and ManPM were evaluated by confocal microscopy using a human in vitro 3D granuloma model, in which the latent phenotype of intracellular *Mtb* was confirmed by Nile Red/Auramine O staining (Figure S2). For confocal imaging, PM and ManPM were fluorescently labeled with RhB (PM-RhB and ManPM-RhB, respectively) to enable visualization and quantification of micelle accumulation. As shown in Figure 3, uptake was quantified as the mean fluorescence intensity (MFI) per granuloma after 2, 4, 6, 24 and 48 h of incubation. Both PM-RhB and ManPM-RhB exhibited a progressive, time-dependent increase in intracellular accumulation throughout the incubation period. At 2 h, no significant differences were observed between the two formulations, indicating comparable initial association with the granuloma. Thereafter, intracellular accumulation increased steadily for both formulations, reflecting continuous micelle internalization by granuloma-associated cells. After 24 h, ManPM-RhB exhibited significantly greater intracellular accumulation than PM-RhB, and this difference was maintained at 48 h, demonstrating that mannose functionalization enhances micelle uptake in the human in vitro latent granuloma model. These findings indicate that mannose functionalization promotes the progressive accumulation of micelles within granuloma-associated cells rather than their initial interaction with the granuloma.

### 3.5. CD206-Expressing Macrophages Represent a Target Population for Mannose-Functionalized Micelles in the Human Granuloma Model

Although enhanced micelle accumulation was observed in the human in vitro 3D granuloma model (Figure 3), the cellular populations responsible for this preferential uptake remained unclear. Flow cytometric characterization demonstrated that the granuloma was enriched in M2-like macrophages (Figure S3), providing a biologically relevant context to investigate whether mannose functionalization preferentially promotes micelle internalization by distinct macrophage subsets. To address this question, human monocyte-derived macrophages were differentiated into M0, M1 and M2 phenotypes and incubated with PM-RhB or ManPM-RhB. Polarization was confirmed by flow cytometry based on CD80/HLA-DR and CD206/DC-SIGN expression for M1 and M2 phenotypes, respectively (Figure S4). Micelle uptake was then quantified by confocal microscopy as the MFI per cell (Figure 4).

To specifically assess the increase in micelle uptake associated with mannose functionalization, the difference in MFI between ManPM-RhB and PM-RhB (ΔMFI) was calculated for each macrophage phenotype. As shown in Figure 4A, the mannose-associated increase in uptake was significantly greater in M2 macrophages compared with M0 and M1 macrophages. To further assess the contribution of baseline uptake of non-functionalized micelles, the MFI profiles of PM-RhB and ManPM-RhB were also evaluated separately across M0, M1, and M2 macrophages and are presented in Supplementary Figure S5.

To further investigate the contribution of mannose-dependent receptor-mediated uptake, macrophages were pretreated with D-mannose to competitively inhibit mannose-dependent binding to CD206. As shown in Figure 4C,E, D-mannose treatment markedly reduced the intracellular accumulation of ManPM-RhB in both M1 and M2-polarized macrophages, with a significantly greater reduction observed in M2 macrophages. Similarly, the percentage inhibition of ManPM-RhB uptake induced by D-mannose was significantly higher in M2 than in M1 macrophages (42.73 ± 4.49% vs. 25.29 ± 7.14%, respectively; paired *t*-test, *p* < 0.05), further supporting a greater contribution of mannose-dependent uptake to ManPM-RhB internalization in M2 macrophages.

Because mannose-dependent uptake was more pronounced in M2 macrophages, we next investigated whether this preferential internalization involved a clathrin-dependent endocytic pathway. As CD206 is a mannose-binding endocytic receptor known to internalize ligands through clathrin-dependent mechanisms, macrophages were pretreated with chlorpromazine (CPZ), a pharmacological inhibitor of clathrin-mediated endocytosis, prior to incubation with PM-RhB or ManPM-RhB (Figure 4D,E). CPZ produced little or no effect on the uptake of either formulation in M1 macrophages. In contrast, CPZ markedly reduced the intracellular accumulation of ManPM-RhB in M2 macrophages, whereas uptake of non-functionalized PM-RhB was minimally affected. Consistent with this differential response, CPZ-induced inhibition of ManPM-RhB uptake was significantly greater in M2 than in M1 macrophages (36.50 ± 5.50% vs. 27.06 ± 7.63%, respectively; paired *t*-test, *p* < 0.05), supporting a greater contribution of clathrin-mediated endocytosis to ManPM-RhB internalization in M2 cells.

Together, these findings further support the involvement of CD206 in the preferential uptake of ManPM-RhB by M2 macrophages. The greater inhibition observed in M2 macrophages following both mannose competition and clathrin inhibition is consistent with the involvement of a CD206-associated, clathrin-dependent pathway in the preferential uptake of mannose-functionalized micelles.

Having established that mannose functionalization promotes preferential uptake by M2 macrophages through a CPZ-sensitive endocytic mechanism, we finally investigated whether this enhanced intracellular delivery translated into improved antimicrobial efficacy against intracellular *Mtb* within the human in vitro latent 3D granuloma model. Seven-day granulomas were treated with free INH, PM-INH or ManPM-INH at concentrations of 0.005, 0.01 and 0.05 μg/mL for either 48 h or 7 days, after which intracellular bacterial survival was determined by CFU counts (Figure 5). Following 48 h of treatment (Figure 5A), both micellar formulations reduced intracellular bacterial burden more efficiently than free INH at the lowest concentrations tested. However, ManPM-INH consistently produced the greatest reduction in CFU counts, particularly at suboptimal drug concentrations, indicating that mannose functionalization enhances intracellular INH drug delivery. After 7 days of treatment (Figure 5B), the differences among formulations became more evident. All formulations reduced intracellular *Mtb* survival in a concentration-dependent manner; however, ManPM-INH showed enhanced antimycobacterial activity compared with free INH and PM-INH, particularly at lower concentrations (0.005 and 0.01 μg/mL INH). At the highest concentration tested, all formulations exhibited a marked reduction in bacterial burden, although ManPM-INH-treated granulomas maintained the lowest CFU values, suggesting an improved intracellular delivery and therapeutic effect of INH through mannose-functionalized micelles. Collectively, these results support a model in which mannose functionalization promotes preferential uptake of polymeric micelles by CD206-expressing M2 macrophages through a clathrin-dependent endocytic pathway, resulting in enhanced intracellular delivery of INH and improved control of intracellular *Mtb* in the human in vitro 3D latent granuloma model.

## 4. Discussion

Tuberculosis granulomas are highly organized and heterogeneous structures in which the cellular composition and functional phenotype of host cells influence both bacterial persistence and therapeutic accessibility. Therefore, the development of effective drug delivery strategies requires considering not only the physicochemical properties of the nanocarrier, but also the biological characteristics of the infected tissue microenvironment. In the present study, our human in vitro granuloma model displayed a predominance of M2-like macrophages, recapitulating a cellular feature previously described in latent TB-associated granulomatous lesions, where alternatively activated macrophage populations are enriched and have been associated with a more permissive environment for persistent *Mtb* [35,36]. From a drug delivery perspective, this observation provided a biologically relevant framework to evaluate whether macrophage-targeted nanocarriers could improve intracellular antimicrobial exposure.

Within this context, our study demonstrates that mannose-functionalized Soluplus^®^ polymeric micelles preferentially accumulated in CD206-expressing macrophages and enhanced intracellular INH activity within the granuloma environment. The mannose receptor (CD206) is widely recognized as an endocytic receptor enriched in alternatively activated macrophage populations and represents an attractive target for macrophage-directed delivery approaches. In addition to its role in cellular uptake, CD206 participates in intracellular trafficking pathways involved in macrophage–pathogen interactions, supporting its potential as a target for different therapeutic strategies against intracellular infections [37]. In our model, mannose functionalization did not simply increase overall nanoparticle uptake, but enhanced micelle accumulation preferentially in M2 macrophages, the predominant macrophage population within the granuloma model. Importantly, this preferential uptake was reduced by both chlorpromazine (CPZ) and D-mannose pretreatment. The effect of CPZ is consistent with the involvement of clathrin-mediated endocytosis, whereas the reduction induced by D-mannose supports a mannose-dependent uptake mechanism involving CD206. We acknowledge that pharmacological inhibitors and competitive sugars are subject to inherent off-target effects, but they nonetheless provide useful indications of internalization mechanisms. Although anti-CD206 blocking antibodies or genetic approaches would provide stronger evidence for receptor-level involvement [38], genetic manipulation of primary human monocyte-derived macrophages remains technically challenging [39]. The greater inhibition observed in M2 macrophages compared with M1 macrophages is consistent with their higher CD206 expression and supports a contribution of mannose-dependent, CD206-associated uptake. However, direct CD206 specificity cannot be established using these approaches alone and warrants further validation with orthogonal strategies. Together, these findings support a model in which CD206-expressing M2 macrophages represent a relevant cellular target for mannose-functionalized micelles.

The relevance of macrophage-targeted delivery in LTBI derives from the central role of macrophages as the primary intracellular niche for *Mtb*. However, macrophages within granulomas are not functionally homogeneous, and their activation state can influence bacterial persistence, inflammatory responses, and therapeutic responsiveness. Although macrophage polarization represents a continuum of functional states rather than discrete phenotypes, alternatively activated M2-like macrophages are generally associated with tissue remodeling, immune regulation, and reduced antimicrobial responses compared with classically activated inflammatory macrophage populations [40,41]. In TB, distinct macrophage phenotypes coexist within granulomatous lesions, and CD206-expressing macrophage populations have been associated with cellular environments that may favor intracellular *Mtb* persistence [35,36]. Therefore, directing INH-loaded micelles toward CD206-expressing macrophages may increase drug exposure within a cellular compartment that is relevant to persistent intracellular bacilli.

Consistent with this rationale, mannose-functionalized micelles showed improved antimycobacterial activity in the human 3D granuloma model, particularly at lower INH concentrations. Importantly, this advantage was not observed to the same extent in axenic cultures, indicating that the improved efficacy was not due to enhanced intrinsic antibacterial activity of INH after encapsulation or mannose functionalization. Rather, the therapeutic benefit appears to result from improved intracellular delivery within the host–cell environment [42]. This distinction is particularly relevant for TB therapy, where achieving sufficient drug exposure at intracellular and tissue sites remains a major challenge. Drug distribution within TB lesions is heterogeneous, and limited penetration into specific cellular and anatomical compartments contributes to incomplete bacterial clearance [17,43]. As highlighted by Gairola et al., macrophage-targeted drug delivery aims to increase intracellular drug accumulation within infected macrophages, thereby enhancing therapeutic efficacy against intracellular *Mtb* [44]. Notably, ManPM-INH showed greater antimycobacterial activity at the lower concentrations tested, particularly 0.005 and 0.01 µg/mL. This concentration range was not intended to reproduce systemic pharmacokinetic exposure, but was deliberately selected as a low-exposure experimental range to investigate formulation efficacy under restricted drug availability, supported by our preliminary MIC determinations and published MIC distributions for INH against *Mtb* [45]. Pulmonary nanomedicine may increase drug exposure at the site of infection while modifying systemic distribution [46]. In addition, mannose-functionalized INH nanocarriers have been shown to improve pulmonary residence and pharmacokinetic parameters following nebulized administration in vivo [47]. However, these studies do not establish that the concentrations evaluated in our in vitro granuloma model correspond to clinically achievable pulmonary exposure. The enhanced activity of ManPM-INH therefore supports improved functional antimycobacterial efficacy under low-exposure conditions, but does not by itself demonstrate increased intracellular INH concentration. Direct intracellular drug quantification, together with pharmacokinetic and in vivo studies, will be required to further establish this mechanism and its translational relevance.

Rather than increasing the intrinsic antimycobacterial activity of INH, these findings indicate that mannose functionalization enhances the pharmacological efficiency of the drug by improving its intracellular delivery to the site of infection. This interpretation is further supported by Clemens et al., who demonstrated that targeted intracellular delivery of antituberculosis drugs increases antimicrobial efficacy compared with equivalent amounts of free drug [48].

From a pharmaceutical perspective, mannose functionalization preserved the physicochemical characteristics of the Soluplus^®^ micellar platform, supporting its suitability for targeted drug delivery. Our previous work demonstrated the potential of Soluplus^®^-based micellar systems to improve the formulation and delivery of antimycobacterial drugs, including rifampicin-loaded micelles with enhanced drug solubilization and delivery properties [49]. Building on this approach, we and others have explored mannose-functionalized nanocarriers as a strategy to enhance macrophage targeting and intracellular antimycobacterial activity, including rifampicin–curcumin nanomicelles and mannose-based INH delivery systems [19,47,50]. The present study extends these previous observations by demonstrating that active targeting can be rationally integrated with the cellular composition of a human granuloma model, providing a biologically relevant framework to evaluate macrophage-directed nanotherapeutic strategies. Particularly, the Soluplus^®^/Gel(man) blend could be interacting via hydrogen bonding, as previously reported [51].

From a pulmonary drug delivery perspective, the nanoscale size of these formulations may also be advantageous for their potential transport through the respiratory mucus layer. In particular, both INH nanoformulations exhibited hydrodynamic diameters below 200 nm, which may be advantageous considering that the mesh size of the mucus network has been reported to range from approximately 100 to 1000 nm [43]. Nevertheless, mucus penetration is influenced not only by particle size but also by surface characteristics and interactions with mucus components. In this way, the neutral zeta potential of our nanoformulations could improve their diffusion through the respiratory mucus barrier, avoiding electrostatic interactions with the negatively charged mucus [52,53].

A major strength of this study is the integration of nanocarrier development with a biologically relevant infection model. Three-dimensional granuloma models provide a valuable platform to investigate host–pathogen interactions and therapeutic strategies under conditions that better reproduce key aspects of TB infection than conventional monocultures [54]. By linking granuloma cellular composition with nanoparticle uptake and antimicrobial activity, our study highlights that successful targeted delivery depends not only on ligand–receptor interactions, but also on the presence of a relevant cellular target within the disease microenvironment [55].

Despite these strengths, several limitations should be considered when interpreting the present findings. First, the study was performed exclusively using in vitro models, and the therapeutic efficacy, pulmonary distribution, pharmacokinetics, and safety of ManPM-INH remain to be evaluated in vivo. Second, the relatively low INH concentrations investigated in the 3D granuloma model were selected as a low-exposure experimental range and were not intended to reproduce systemic or pulmonary pharmacokinetic exposure; therefore, their translational relevance remains to be established. Third, although the use of primary human cells provides biological relevance, donor-to-donor variability may influence macrophage phenotype, nanoparticle uptake, and antimicrobial responses. Finally, the translational development of the gelatin–mannose conjugate requires further evaluation of manufacturing scalability, formulation reproducibility, and immunological safety, particularly in the context of repeated pulmonary administration. Although gelatin is widely used in biomedical applications because of its favorable biocompatibility and biodegradability [56], the immunological safety of the specific gelatin–mannose conjugate used here has not been established. Recombinant human gelatin may represent a potential alternative to animal-derived gelatin because it can address concerns related to allergic reactions and variability associated with animal-derived materials [57]. Nevertheless, the suitability and safety of such alternatives for pulmonary administration would require dedicated preclinical evaluation [58].

Taken together, these findings support the potential of PM-INH and ManPM-INH as nanotechnological platforms for pulmonary INH delivery. Importantly, the incorporation of the mannose-functionalized coating did not compromise the colloidal stability of the system following nebulization. Thus, ManPM-INH combines suitable nanoscale physicochemical characteristics with the potential for macrophage-targeted pulmonary delivery.

Overall, these findings support mannose-functionalized Soluplus^®^ micelles as a rational macrophage-targeted platform for intracellular INH delivery against *Mtb*. The predominance of a CD206-expressing M2-like macrophage population in the human granuloma model, consistent with features described in latent tuberculosis lesions, provided a biologically relevant context to evaluate mannose-mediated targeting. Under these conditions, mannose functionalization enhanced intracellular micelle accumulation and translated into improved antimycobacterial activity. These results emphasize the importance of considering granuloma cellular architecture when designing next-generation nanotherapeutic strategies for TB.

## Figures and Tables

**Figure 1:**
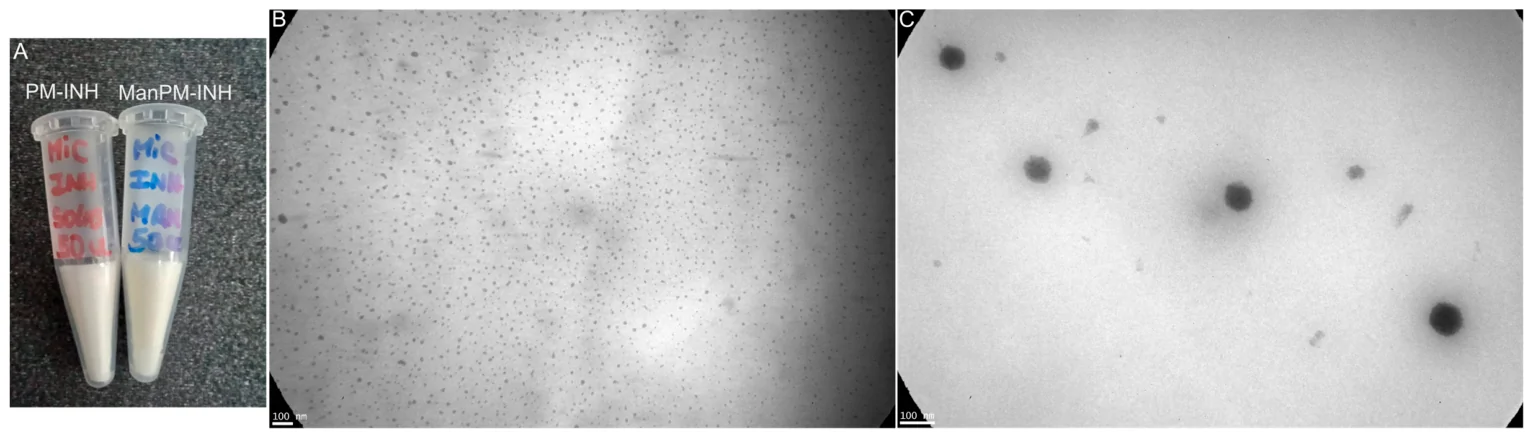
(**A**) Macroscopic aspect of freeze-dried PM-INH and ManPM-INH. (**B**,**C**) Magnification of TEM micrographs of PM-INH and ManPM-INH, respectively. Bar Scale: 100nm.

**Figure 2:**
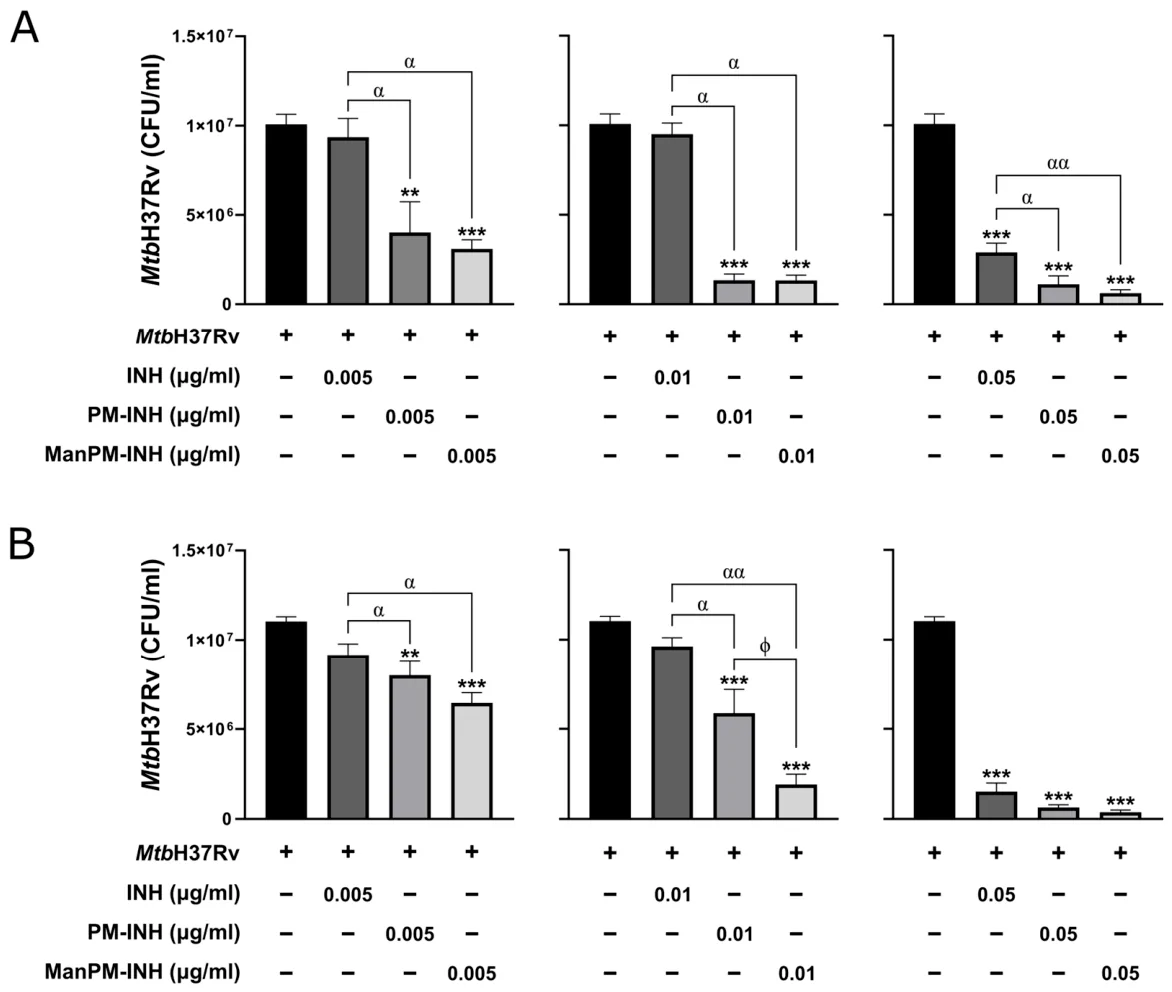
Colony-forming units (CFU) counts of *Mtb*H37Rv cultures treated with INH-PM formulations. *Mtb*H37Rv bacterial cultures were treated with different concentrations of INH, PM-INH, or ManPM-INH (0.005–0.05 μg/mL) for either (**A**) 48 h or (**B**) 7 days. Bars represent CFU Mean ± standard error of the mean (SEM) from N = 7 independent experiments. ** *p* < 0.01, *** *p* < 0.001 compared to the *Mtb*H37Rv untreated control. α condition vs. *Mtb*H37Rv treated with the same concentration of soluble INH, *p* < 0.05, αα *p* < 0.01. ϕ condition vs. *Mtb*H37Rv treated with the same concentration of PM-INH, *p* < 0.05. One-way ANOVA with post hoc Tukey’s multiple comparisons test.

**Figure 3:**
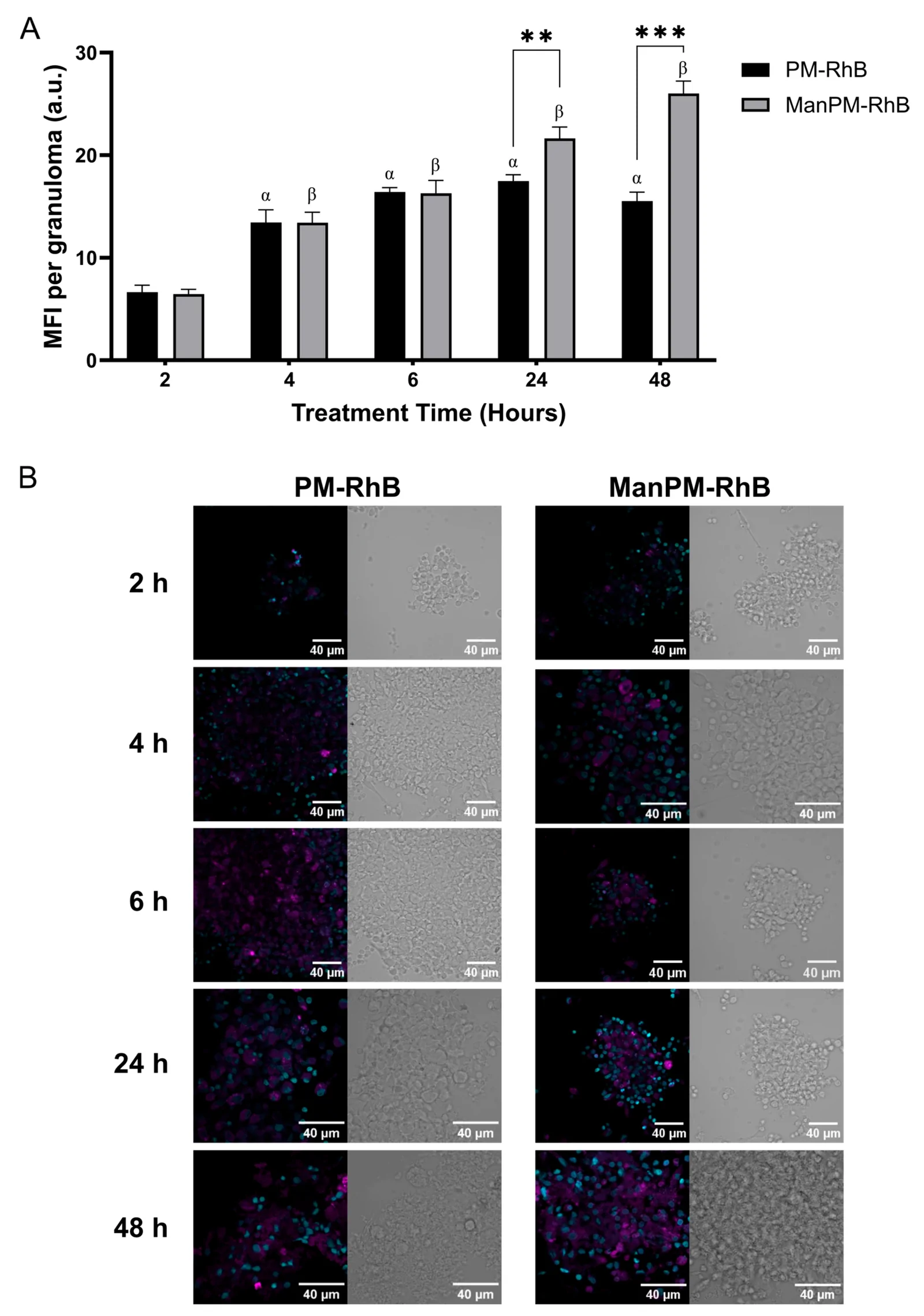
Uptake kinetics of rhodamine B-labeled polymeric micelles (PM-RhB) and rhodamine B-labeled mannosylated polymeric micelles (ManPM-RhB) in the in vitro latency granuloma model. (**A**) Internalization was assessed by confocal microscopy after incubation periods ranging from 2 to 48 h. (**B**) Representative images depict DAPI-stained nuclei (cyan) and rhodamine-loaded micelles (magenta) from individual in vitro granulomas. Data represents the mean rhodamine fluorescence intensity per granuloma, arbitrary units (a.u.) ± SEM of N = 7 independent healthy donors. α condition vs. 2 h PM-RhB, *p* < 0.05; β condition vs. 2 h ManPM-RhB, ** *p* < 0.01, *** *p* < 0.001 within the same time of treatment. Two-way ANOVA with post hoc Tukey’s multiple comparisons test.

**Figure 4:**
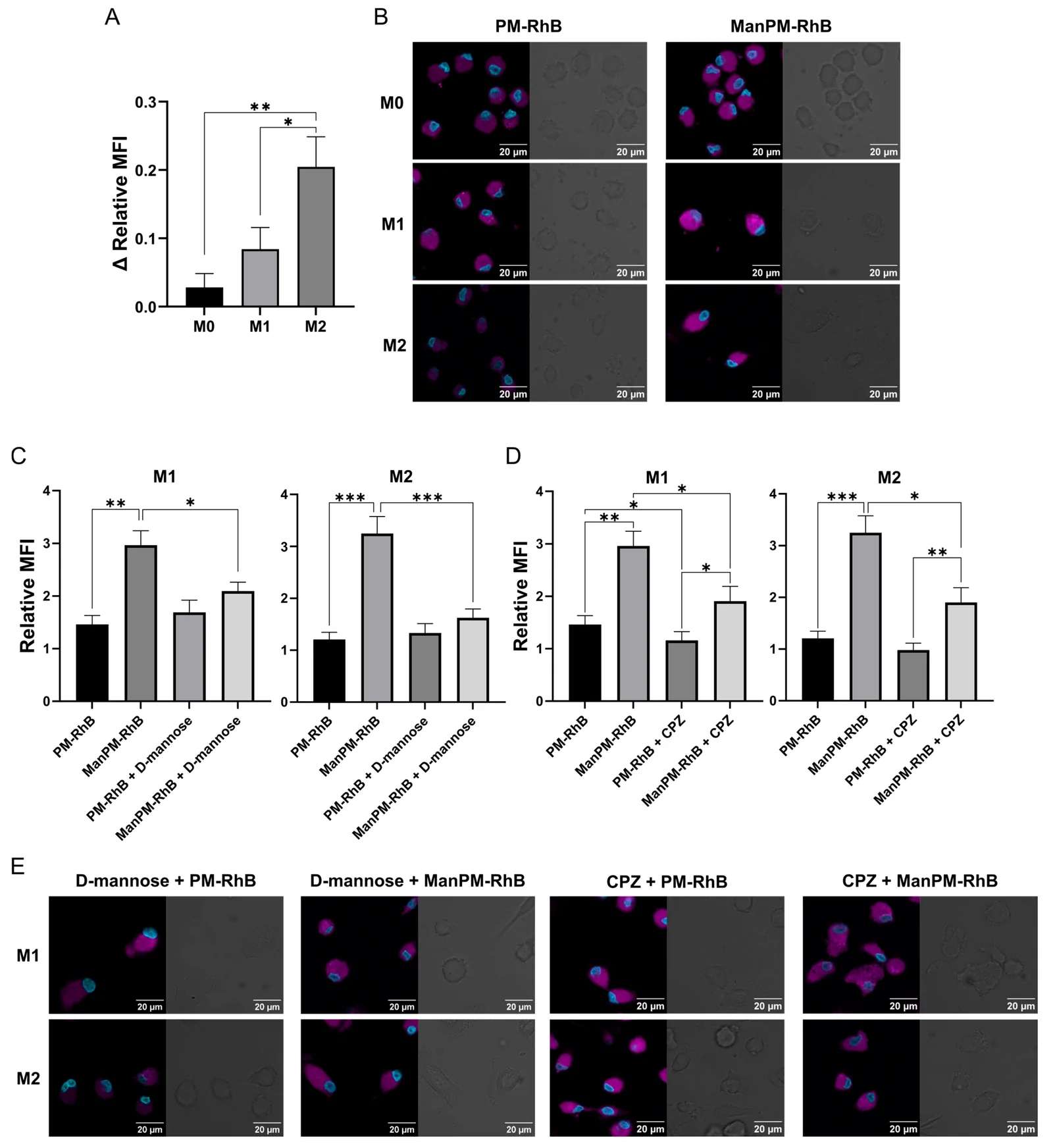
Uptake of PM-RhB and ManPM-RhB by M0, M1, and M2 macrophages. (**A**) Data show the difference (Δ) in relative mean fluorescence intensity (MFI) between macrophages treated with ManPM-RhB and treated with PM-RhB within the same experiment (ManPM-RhB MFI minus PM-RhB MFI). (**C**) Macrophages were pretreated with D-mannose (50 mM). Internalization was assessed as MFI per cell. (**D**) Macrophages were pretreated with CPZ (15 µM) prior to incubation with PM-RhB or ManPM-RhB. (**B**,**E**) Representative confocal images show cell nuclei stained with DAPI (cyan) and rhodamine-loaded micelles (magenta). Data are presented as mean ± SEM of N = 6 independent healthy donors. * *p* < 0.05, ** *p* < 0.01, *** *p* < 0.001. Statistical analysis was performed using a one-way repeated-measures ANOVA with Greenhouse–Geisser correction, followed by Holm–Šidák multiple-comparisons test.

**Figure 5:**
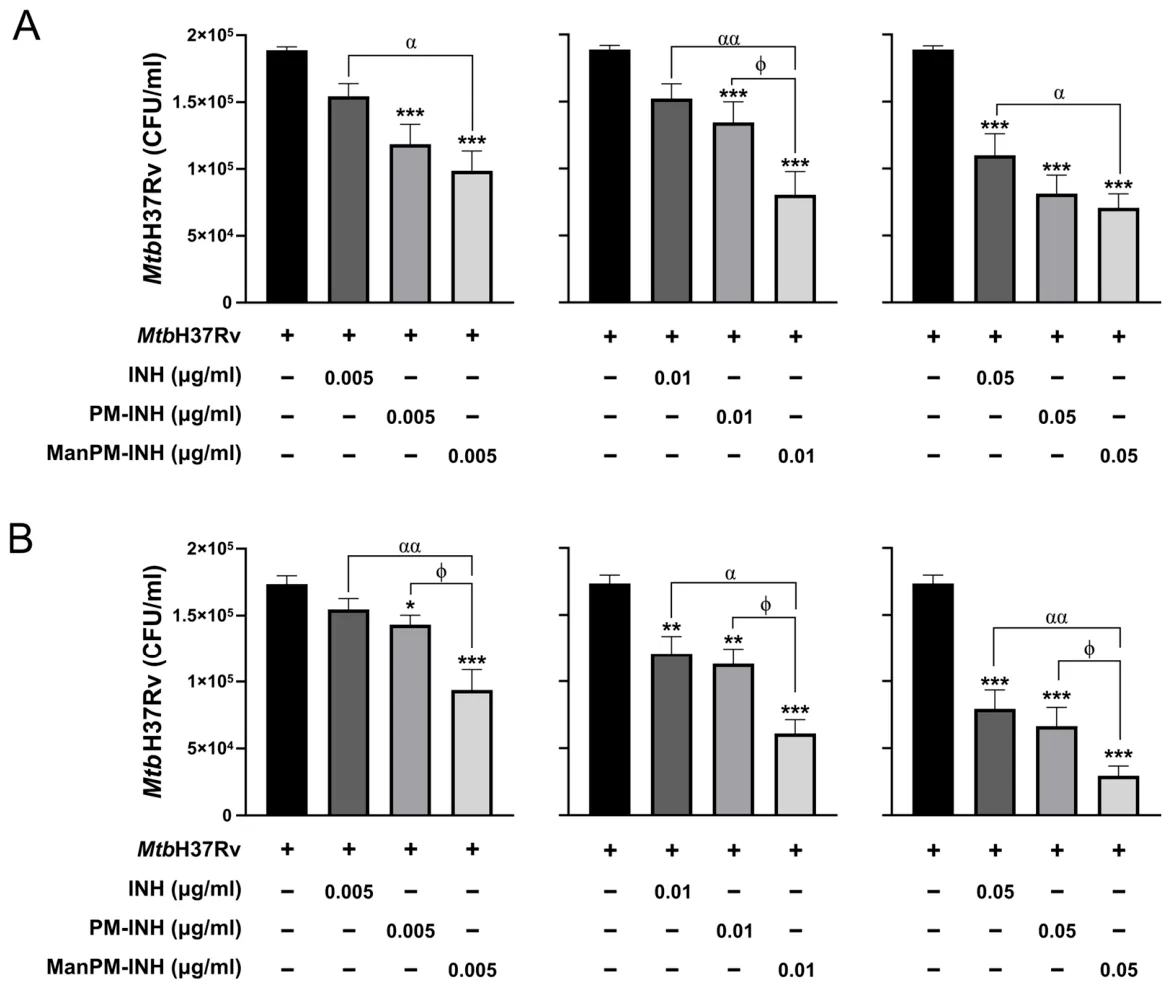
Intracellular survival of dormant *Mtb*H37Rv in the in vitro granuloma model. After 7 days of granuloma formation, cultures were treated with INH, PM-INH, or ManPM-INH at concentrations of 0.005, 0.01, and 0.05 µg/mL for 48 h (**A**) or 7 days (**B**). Bars represent CFU Mean ± SEM from N = 7 independent healthy donors. * *p* < 0.05, ** *p* < 0.01, *** *p* < 0.001 compared to the *Mtb*H37Rv untreated control. α condition vs. *Mtb*H37Rv treated with the same concentration of soluble INH, *p* < 0.05, αα *p* < 0.01. ϕ condition vs. *Mtb*H37Rv treated with the same concentration of INH-PM, *p* < 0.05. One-way ANOVA with post hoc Tukey’s multiple comparisons test.

**Table 1:** Micellar size and size distribution of PM-INH and ManPM-INH before and after freeze-drying and redispersion in distilled water at 25 °C. Results are expressed as the mean (N = 3) ± standard deviation (S.D.).

Sample	Size (nm) (±S.D.)	
	Z-Ave	PDI
PM-INH	115.1 (1.9)	0.214 (0.003)
Freeze-dried PM-INH	118.3 (3.4)	0.219 (0.015)
ManPM-INH	166.5 (1.5)	0.403 (0.013)
Freeze-dried ManPM-INH	131.2 (0.2)	0.240 (0.006)

**Table 2:** Micellar size and size distribution of PM-INH and ManPM-INH diluted (1/5) in distilled water and normal saline solution (NaCl 0.9% *w*/*v*) before and after in vitro nebulization at 25 °C. Results are expressed as the mean (n = 3) ± standard deviation (S.D.).

Sample	Dilution Medium	In Vitro Nebulization	Size (nm) (±S.D.)	
			Z-Ave	PDI (±S.D.)
PM-INH	Normal saline solution	−	75.4 (0.8)	0.056 (0.017)
		√	75.5 (0.5)	0.064 (0.010)
ManPM-INH		−	76.8 (0.3)	0.058 (0.012)
		√	75.2 (0.3)	0.075 (0.009)
PM-INH	Distilled water	−	80.0 (0.2)	0.136 (0.018)
		√	75.5 (0.3)	0.053 (0.015)
ManPM-INH		−	78.1 (0.8)	0.064 (0.027)
		√	76.9 (0.7)	0.046 (0.010)

## Data Availability

The raw data supporting the conclusions of this article will be made available by the authors on request.

## Supplementary Materials

The following supporting information can be downloaded at: https://www.mdpi.com/article/10.3390/pharmaceutics18101279/s1. Figure S1: Viability of PBMCs after exposure to isoniazid-free Soluplus^®^ polymeric micelles (PM), isoniazid-free mannosylated Soluplus^®^ polymeric micelles (ManPM), isoniazid-loaded Soluplus^®^ polymeric micelles (PM-INH), and isoniazid-loaded mannosylated Soluplus^®^ polymeric micelles (ManPM-INH), as determined by XTT assay. PBMCs were incubated with the different formulations at concentrations ranging from 0.003 to 0.050 µg/mL for 48 h (A–B) or 7 days (C–D). Cell viability remained above 70% in all conditions. Dashed lines indicate the 70% threshold. Data are presented as the mean ± SEM (N = 4). Figure S2: *Mtb* within the granuloma goes into a dormant state. Auramine-O (green) and Nile Red (red) staining of granuloma sections shows Nile Red-positive *Mtb* cells within the granuloma. White arrows point out Nile Red-positive sections of the bacteria. Figure S3: Proportion of M1 and M2 macrophages within in vitro granulomas, as determined by flow cytometry. M1 macrophages were defined as CD80^+^HLA-DR^+^ cells, whereas M2 macrophages were defined as CD206^+^DC-SIGN^+^ cells. Data are shown as the mean percentage of CD14^+^ cells for each cellular profile ± SEM of N = 4 independent experiments. * *p* < 0.05, One-way ANOVA with post hoc Tukey’s multiple comparisons test. Figure S4: Expression of surface markers in M0, M1, and M2 macrophages. Surface expression of HLA-DR (A), CD80 (B), CD206 (C), and DC-SIGN (D) was determined by flow cytometry. Results are expressed as mean fluorescence intensity (MFI) relative to autofluorescence ± SEM from six independent healthy donors (N = 6). M1 macrophages showed higher expression of HLA-DR and CD80, while M2 macrophages exhibited increased CD206 and DC-SIGN expression. * *p* < 0.05, ** *p* < 0.01. Statistical analysis was performed using a one-way repeated-measures ANOVA with Greenhouse–Geisser correction, followed by Tukey’s multiple-comparisons test. Figure S5: Relative MFI profiles of PM-RhB and ManPM-RhB uptake by M0, M1, and M2 macrophages. Relative mean fluorescence intensity (MFI) of PM-RhB and ManPM-RhB in M0, M1, and M2 macrophages. For each donor, at least 10 individual cells were analyzed per phenotype and formulation. Cell-level MFI values were averaged to obtain one value per donor and experimental condition. Data are presented as mean ± SEM from six independent healthy donors (N = 6). ** *p* < 0.01, *** *p* < 0.001. Statistical analysis was performed using repeated-measures ANOVA with Greenhouse–Geisser correction, followed by Holm–Šidák multiple-comparisons test.

## Abbreviations

The following abbreviations are used in this manuscript:

LTBI	Latent Tuberculosis Infection
TB	Tuberculosis
*Mtb*	*Mycobacterium tuberculosis*
INH	Isoniazid
PM	Polymeric Micelles
ManPM	Mannose-functionalized Polymeric Micelles
PM-INH	INH-loaded Polymeric Micelles
ManPM-INH	INH-loaded Mannose-functionalized Polymeric Micelles
RhB	Rhodamine B
PM-RhB	Rhodamine B-loaded Polymeric Micelles
ManPM-RhB	Rhodamine B-loaded Mannose-functionalized Polymeric Micelles
CFU	Colony-Forming Unit
3D	Three-Dimensional
PDI	Polydispersity Index
DLS	Dynamic Light Scattering
Z-ave	Z-average
Gel(man)	Mannose-modified Gelatin
OADC	Oleic acid–albumin–dextrose–catalase
