# Supplementary material for: Mannose-Functionalized Polymeric Micelles Target CD206-Expressing Macrophages Within Human Tuberculosis Granulomas and Improve Isoniazid Delivery
Source: Pharmaceutics. 2026 Oct 9;18(10):1279. doi: 10.3390/pharmaceutics18101279 (PMC13708602; doi:10.3390/pharmaceutics18101279)
Supplement: Supplementary file 1 [file pharmaceutics-18-01279-s001.zip › pharmaceutics-4561527-supplementary.pdf]

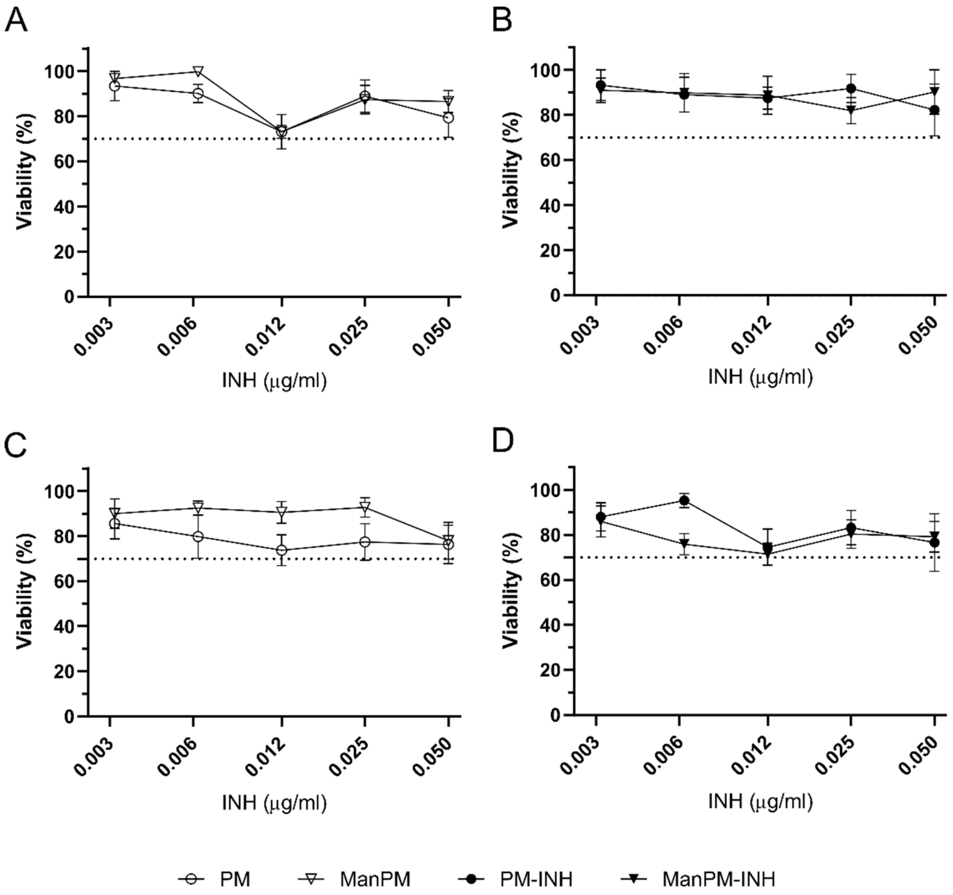

Figure S1. Viability of PBMCs after exposure to isoniazid-free Soluplus® polymeric micelles (PM), isoniazid-free mannosylated Soluplus® polymeric micelles (ManPM), isoniazid-loaded Soluplus® polymeric micelles (PM-INH), and isoniazid-loaded mannosylated Soluplus® polymeric micelles (ManPM-INH), as determined by XTT assay. PBMCs were incubated with the different formulations at concentrations ranging from 0.003 to 0.050 µg/mL for 48 h (**A**–**B**) or 7 days (**C**–**D**). Cell viability remained above 70% in all conditions. Dashed lines indicate the 70% threshold. Data are presented as the mean ± SEM (N = 4).

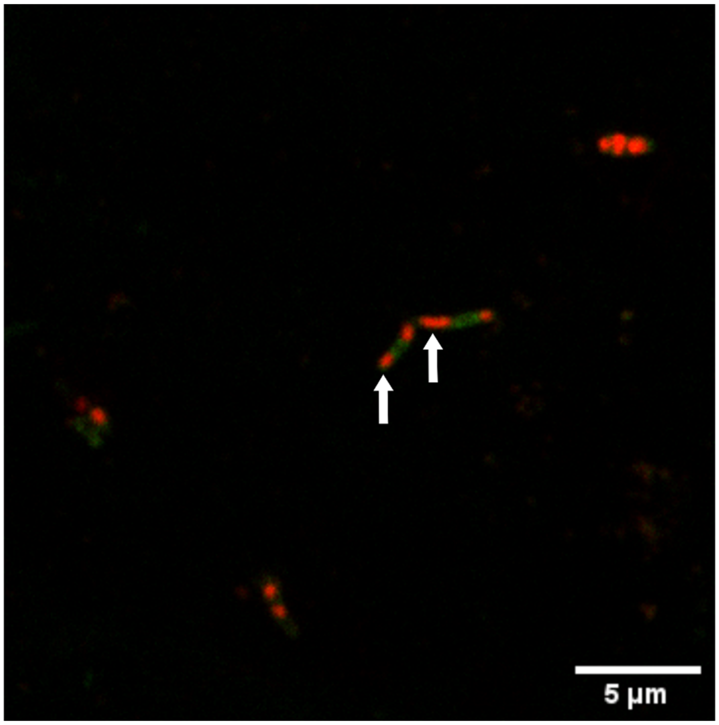

Figure S2. *Mtb* within the granuloma goes into a dormant state. Auramine-O (green) and Nile Red (red) staining of granuloma sections shows Nile Red-positive *Mtb* cells within the granuloma. White arrows point out Nile Red-positive sections of the bacteria.

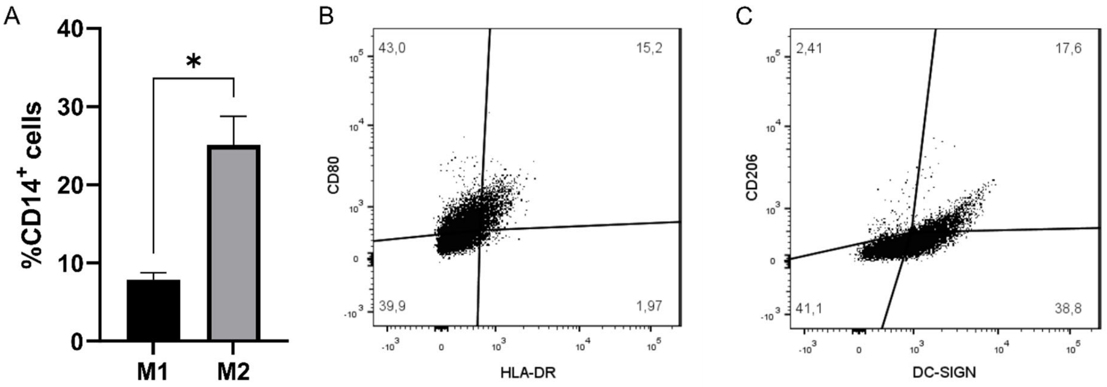

Figure S3. Proportion of M1 and M2 macrophages within in vitro granulomas, as determined by flow cytometry. M1 macrophages were defined as CD80$^+$HLA-DR$^+$ cells, whereas M2 macrophages were defined as CD206$^+$DC-SIGN$^+$ cells. Data are shown as the mean percentage of CD14$^+$ cells for each cellular profile ± SEM of N = 4 independent experiments. **p*<0.05, One-way ANOVA with post hoc Tukey's multiple comparisons test.

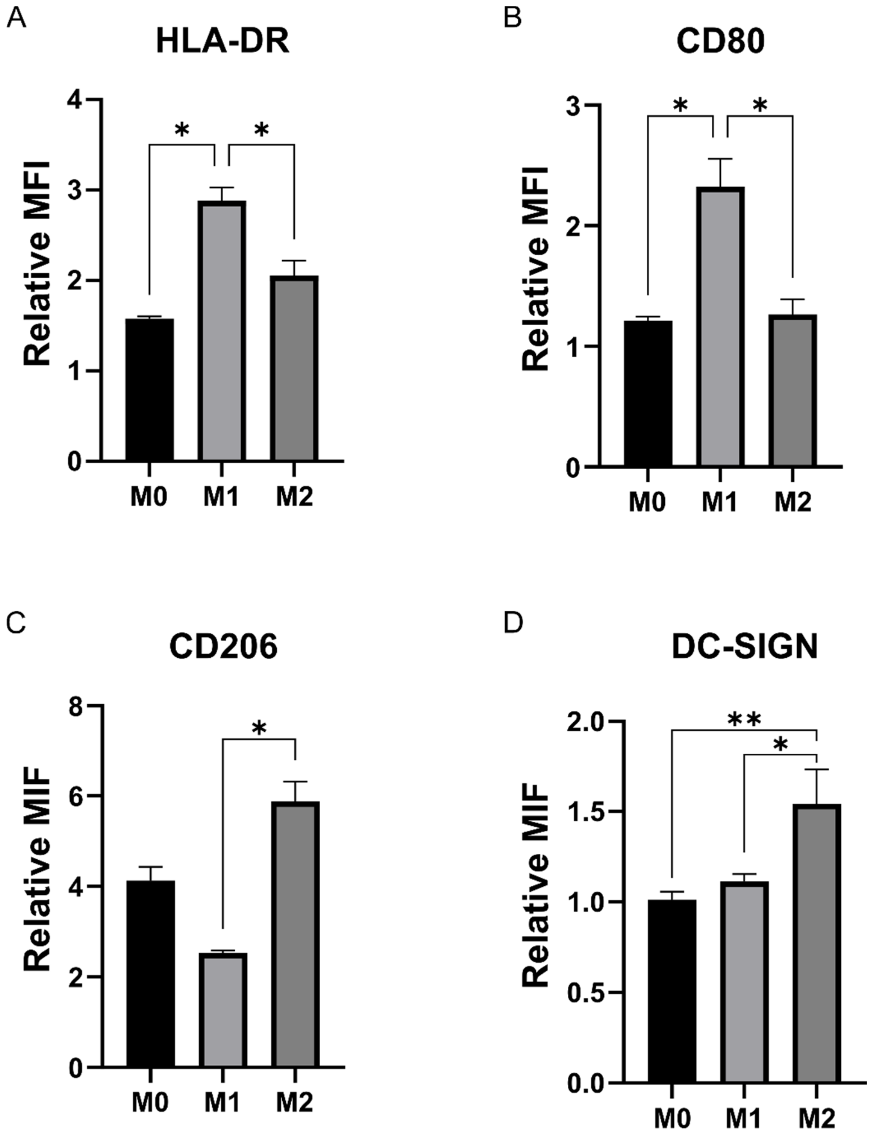

Figure S4. Expression of surface markers in M0, M1, and M2 macrophages. Surface expression of HLA-DR (**A**), CD80 (**B**), CD206 (**C**), and DC-SIGN (**D**) was determined by flow cytometry. Results are expressed as mean fluorescence intensity (MFI) relative to autofluorescence ± SEM from six independent healthy donors (N = 6). M1 macrophages showed higher expression of HLA-DR and CD80, while M2 macrophages exhibited increased CD206 and DC-SIGN expression. *$p < 0.05$, **$p < 0.01$. Statistical analysis was performed using a one-way repeated-measures ANOVA with Greenhouse–Geisser correction, followed by Tukey's multiple-comparisons test.

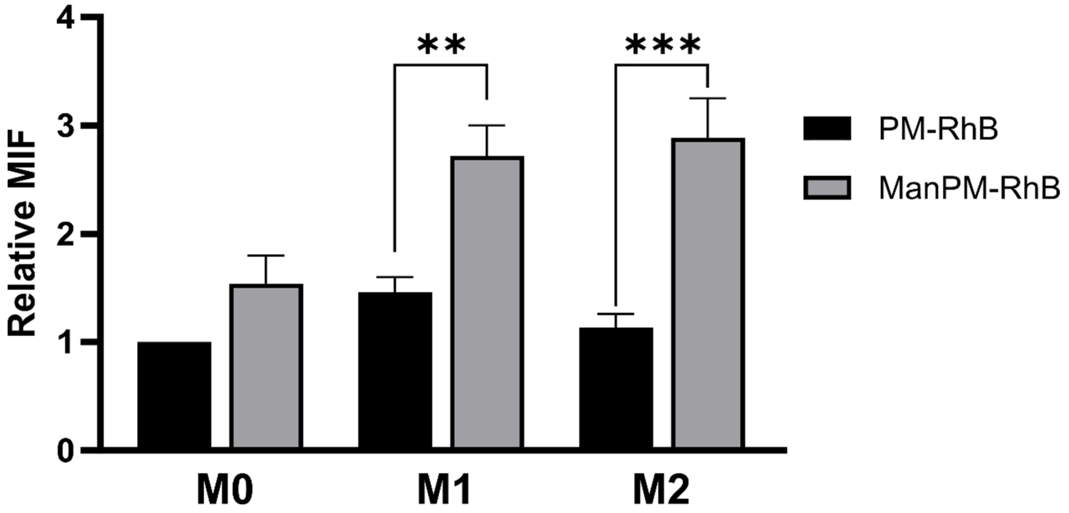

Figure S5. Relative MFI profiles of PM-RhB and ManPM-RhB uptake by M0, M1, and M2 macrophages. Relative mean fluorescence intensity (MFI) of PM-RhB and ManPM-RhB in M0, M1, and M2 macrophages. For each donor, at least 10 individual cells were analyzed per phenotype and formulation. Cell-level MFI values were averaged to obtain one value per donor and experimental condition. Data are presented as mean ± SEM from six independent healthy donors (N = 6). **$p < 0.01$, ***$p < 0.001$. Statistical analysis was performed using repeated-measures ANOVA with Greenhouse–Geisser correction, followed by Holm–Šidák multiple-comparisons test.
